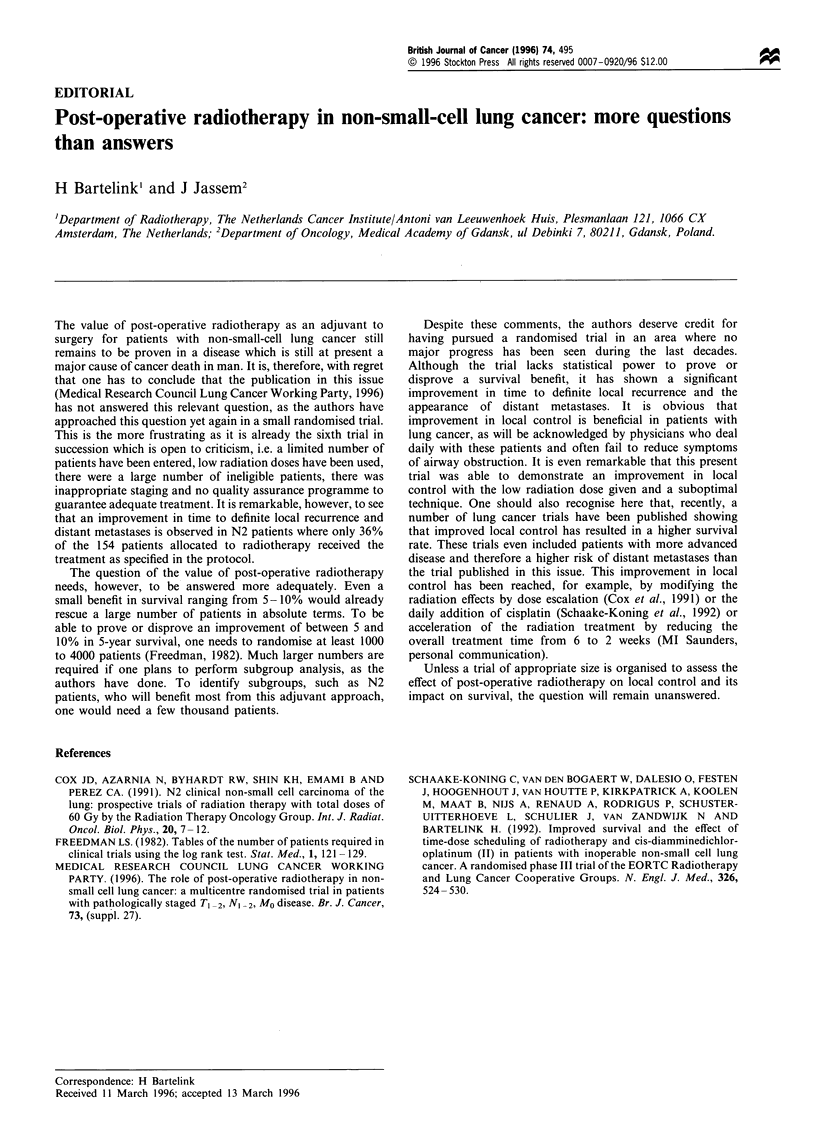# Post-operative radiotherapy in non-small-cell lung cancer: more questions than answers.

**DOI:** 10.1038/bjc.1996.391

**Published:** 1996-08

**Authors:** H. Bartelink, J. Jassem


British Journal of Cancer (1996) 74, 495
© 1996 Stockton Press All rights reserved 0007-0920/96 $12.00

EDITORIAL

### Post-operative radiotherapy in non-small-cell lung cancer: more questions than answers

H Bartelink[1] and J Jassem[2]

[1]*Department of Radiotherapy, The Netherlands Cancer Institute/Antoni van Leeuwenhoek Huis, Plesmanlaan 121, 1066 CX Amsterdam, The Netherlands;* [2]*Department of Oncology, Medical Academy of Gdansk, ul Debinki 7, 80211, Gdansk, Poland.*

The value of post-operative radiotherapy as an adjuvant to surgery for patients with non-small-cell lung cancer still remains to be proven in a disease which is still at present a major cause of cancer death in man. It is, therefore, with regret that one has to conclude that the publication in this issue (Medical Research Council Lung Cancer Working Party, 1996) has not answered this relevant question, as the authors have approached this question yet again in a small randomised trial. This is the more frustrating as it is already the sixth trial in succession which is open to criticism, i.e. a limited number of patients have been entered, low radiation doses have been used, there were a large number of ineligible patients, there was inappropriate staging and no quality assurance programme to guarantee adequate treatment. It is remarkable, however, to see that an improvement in time to definite local recurrence and distant metastases is observed in N2 patients where only 36% of the 154 patients allocated to radiotherapy received the treatment as specified in the protocol.

The question of the value of post-operative radiotherapy needs, however, to be answered more adequately. Even a small benefit in survival ranging from 5–10% would already rescue a large number of patients in absolute terms. To be able to prove or disprove an improvement of between 5 and 10% in 5-year survival, one needs to randomise at least 1000 to 4000 patients (Freedman, 1982). Much larger numbers are required if one plans to perform subgroup analysis, as the authors have done. To identify subgroups, such as N2 patients, who will benefit most from this adjuvant approach, one would need a few thousand patients.

Despite these comments, the authors deserve credit for having pursued a randomised trial in an area where no major progress has been seen during the last decades. Although the trial lacks statistical power to prove or disprove a survival benefit, it has shown a significant improvement in time to definite local recurrence and the appearance of distant metastases. It is obvious that improvement in local control is beneficial in patients with lung cancer, as will be acknowledged by physicians who deal daily with these patients and often fail to reduce symptoms of airway obstruction. It is even remarkable that this present trial was able to demonstrate an improvement in local control with the low radiation dose given and a suboptimal technique. One should also recognise here that, recently, a number of lung cancer trials have been published showing that improved local control has resulted in a higher survival rate. These trials even included patients with more advanced disease and therefore a higher risk of distant metastases than the trial published in this issue. This improvement in local control has been reached, for example, by modifying the radiation effects by dose escalation (Cox *et al.*, 1991) or the daily addition of cisplatin (Schaake-Koning *et al.*, 1992) or acceleration of the radiation treatment by reducing the overall treatment time from 6 to 2 weeks (MI Saunders, personal communication).

Unless a trial of appropriate size is organised to assess the effect of post-operative radiotherapy on local control and its impact on survival, the question will remain unanswered.

### References

COX JD, AZARNIA N, BYHARDT RW, SHIN KH, EMAMI B AND PEREZ CA. (1991). N2 clinical non-small cell carcinoma of the lung: prospective trials of radiation therapy with total doses of 60 Gy by the Radiation Therapy Oncology Group. *Int. J. Radiat. Oncol. Biol. Phys.*, **20,** 7–12.

FREEDMAN LS. (1982). Tables of the number of patients required in clinical trials using the log rank test. *Stat. Med.*, **1,** 121–129.

MEDICAL RESEARCH COUNCIL LUNG CANCER WORKING PARTY. (1996). The role of post-operative radiotherapy in non-small cell lung cancer: a multicentre randomised trial in patients with pathologically staged $T_{1-2}$, $N_{1-2}$, $M_0$ disease. *Br. J. Cancer*, **73,** (suppl. 27).

SCHAAKE-KONING C, VAN DEN BOGAERT W, DALESIO O, FESTEN J, HOOGENHOUT J, VAN HOUTTE P, KIRKPATRICK A, KOOLEN M, MAAT B, NIJS A, RENAUD A, RODRIGUS P, SCHUSTER-UITTERHOEVE L, SCHULIER J, VAN ZANDWIJK N AND BARTELINK H. (1992). Improved survival and the effect of time-dose scheduling of radiotherapy and cis-diamminedichloroplatinum (II) in patients with inoperable non-small cell lung cancer. A randomised phase III trial of the EORTC Radiotherapy and Lung Cancer Cooperative Groups. *N. Engl. J. Med.*, **326,** 524–530.

Correspondence: H Bartelink
Received 11 March 1996; accepted 13 March 1996